# Microwave-Assisted Resolution of α-Lipoic Acid Catalyzed by an Ionic Liquid Co-Lyophilized Lipase

**DOI:** 10.3390/molecules20069949

**Published:** 2015-05-29

**Authors:** Ning Liu, Lei Wang, Zhi Wang, Liyan Jiang, Zhuofu Wu, Hong Yue, Xiaona Xie

**Affiliations:** 1 Central Laboratory, The Second Hospital of Jilin University, Changchun 130021, China; E-Mail: liu_ning@jlu.edu.cn; 2 Key Laboratory of Molecular Enzymology and Engineering of Ministry of Education, College of Life Science, Jilin University, Changchun 130012, China; E-Mails: w_lei@jlu.edu.cn (L.W.); wangzhi@jlu.edu.cn (Z.W.); jiangliyan@jlu.edu.cn (L.J.); wuzf06@mails.jlu.edu.cn (Z.W.); xinqingtianlan@sina.cn (H.Y.); 3 The First Hospital of Jilin University, Changchun 130021, China

**Keywords:** ionic liquid co-lyophilized lipase, α-lipoic acid, enantioselectivity, enzyme activity, esterification, microwave

## Abstract

The combination of the ionic liquid co-lyophilized lipase and microwave irradiation was used to improve enzyme performance in enantioselective esterification of α-lipoic acid. Effects of various reaction conditions on enzyme activity and enantioselectivity were investigated. Under optimal condition, the highest enantioselectivity (*E* = 41.2) was observed with a high enzyme activity (178.1 μmol/h/mg) when using the ionic liquid co-lyophilized lipase with microwave assistance. Furthermore, the ionic liquid co-lyophilized lipase exhibited excellent reusability under low power microwave.

## 1. Introduction

As an important cofactor involved in many enzyme-catalyzed reactions [1], α-lipoic acid has already been used to treat many diseases [2,3,4]. Since (*R*)-α-lipoic acid exhibits higher biological activity than its (*S*)-enantiomer [5], great attention has been focused on the stereoselective synthesis of its (*R*)-enantiomer. There are two main routes to synthesize (*R*)-α-lipoic acid, including the chemical synthesis and biocatalytic process. The chemical synthesis is usually conducted using asymmetric synthesis [6,7] or synthesis from a chiral starting material [8]. Due to the interest in a product with high enantiomeric purity, several alternative biocatalytic processes have been developed recently, including reductase catalysis [9,10,11,12], Bakers’ yeast reduction [13], and lipase-catalyzed kinetic resolution [14,15,16,17]. Among these processes, lipase-catalyzed kinetic resolution is one of the most attractive methods. Zhou and his group had successfully prepared the chiral chlorohydrin precursor of (*R*)-α-lipoic acid via lipase catalyzed enantioselective transesterification [17]. In 1997, Fadnavis firstly reported that the commercially available *Candida rugosa* lipase can catalyze the enzymatic resolution of α-lipoic acid directly [14]. Yan and his co-workers have reported that the lipase from *Aspergillus oryzae* WZ007 is another potential candidate for enzymatic resolution of α-lipoic acid [15,16]. However, all these lipases exhibited poor enzyme performance (low enzyme activity or unsatisfied enantioselectivity), which may increase the reaction time and reduce the yield of the enantiomerically pure product. Thus, novel enzymes and new techniques are warranted to improve the performance of enantioselective esterification.

Microwave irradiation (MW) and ionic liquid (IL) are two important and rapid developing technologies in green chemistry [18,19,20,21,22,23,24,25]. The combination of MW and IL has already attracted a great deal of attention for enzymatic reactions in recent years [26,27,28]. In reports, IL was used as reaction media due to its excellent microwave-absorbing ability. However, the high viscosity of IL may have an adverse effect on the mass transfer of the substrates and worsen enzyme performance [29]. It is well known that a small amount of IL can induce dramatic changes in the overall dielectric properties of the reaction medium under microwave irradiation [30,31]. Furthermore, lyophilization of enzyme in the presence of a small amount of IL can improve the activity, stability, and enantioselectivity of enzymes [32,33,34,35,36,37]. All of the above reports may provide a new breakthrough to improve the activity and enantioselectivity of enzyme during biocatalytic synthesis.

**Scheme 1:**
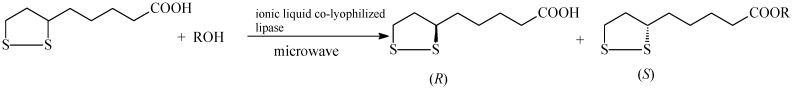
Enantioselective esterification of α-lipoic acid catalyzed by ionic liquid co-lyophilized lipase under microwave.

In the present work, the combination of the ionic liquid co-lyophilized lipase and microwave irradiation was used to improve the enzyme performance in the enantioselective esterification of α-lipoic acid. The enzyme has been firstly co-lyophilized with a small amount of ionic liquid and then used as catalyst to carry out the enantioselective esterification of α-lipoic acid under microwave irradiation (Scheme 1). The effects of reaction conditions on the activity and enantioselectivity were investigated and the reusability of the ionic liquid co-lyophilized lipase under microwave was also studied.

## 2. Results and Discussion

### 2.1. Effect of Lipase Source

Four commercially available lipases were selected for enantioselective esterification of α-lipoic acid and the results were listed in Table 1. All selected lipases could catalyze the enantioselective esterification of α-lipoic acid. Moreover, all the enzymes favor the (*S*)-enantiomer of α-lipoic acid, but exhibited poor enantioselectivity. Among the tested lipases, CLL (*Candida lipolytic* lipase) showed the highest enantioselectivity (*E* = 3.2) and a higher enzyme activity (3.7 μmol/h/mg). Thus, CLL was selected as the catalyst for further study.

**Table 1:** Effect of lipase source on the enantioselective esterification of α-lipoic acid.

Lipase	Reaction Time (h)	Conversion (%)	*ee*_s_ (%)	Enzyme Activity (μmol/h/mg)	Enantioselectivity ( *E* value)	Stereoselectivity
*Candida cylindracea* A.Y. lipase (AYL)	12	19.8	8.4	3.3	2.2	*S*
*Mucor miehei* lipase (MML)	20	24.0	6.3	2.4	1.6	*S*
*Porcine pancreatic* lipase (PPL)	30	16.5	5.8	1.1	1.9	*S*
*Candida lipolytic* lipase (CLL)	12	22.2	13.6	3.7	3.2	*S*

Reaction conditions: (*R*,*S*)-α-lipoic acid (2 mmol), lipase (10 mg), *n*-octanol (5 mmol) and heptane (10 mL) were performed at 30 °C and 150 rpm.

### 2.2. Effect of Organic Media

Organic media can alter the solubility of substrates and affect enzyme performance [38]. In this study, organic solvents with different log *P* were selected to investigate the effect of the reaction medium; log *P* is the most frequently used parameter to denote the polarity or hydrophobicity of a solvent [39]. The results shown in Table 2 indicate that the best catalytic performance of CLL was observed when cyclohexane was used as the reaction media. When polar solvents were used as the reaction media, they could disrupt the functional structure of the enzyme by stripping off the essential water from the protein and decrease the enzyme activity and the enantioselectivity [40,41].

Other reaction conditions were also investigated (data not shown). Our experimental results indicated that *n*-octanol was the most suitable acyl-acceptor. The optimal substrate molar ratio (α-lipoic acid/*n*-octanol) was 1:2.5 and the enzyme dosage in this reaction system was 10 mg.

**Table 2:** Effect of organic solvents on the enantioselective esterification of α-lipoic acid.

Solvent	Log *P*	Reaction Time (h)	Conversion (%)	*ee*_s_ (%)	Enzyme Activity (μmol/h/mg)	Enantioselectivity (*E* value)
Isooctane	4.5	12	25.2	3.0	4.2	1.2
Heptane	4.0	12	22.2	13.6	3.7	3.2
Cyclohexane	3.2	12	21.6	17.5	3.6	5.3
Toluene	2.5	15	15.8	8.5	2.1	2.9
Acetonitrile	−0.33	28	19.6	9.3	1.4	2.4
1,4-Dioxane	−1.1	60	26.8	4.2	0.9	1.3

Reaction conditions: (*R*,*S*)-α-lipoic acid (2 mmol), CLL (10 mg), *n*-octanol (5 mmol) and organic solvent (10 mL) were performed at 30 °C and 150 rpm.

### 2.3. Effect of ILs Used for the Ionic Liquid Co-Lyophilized Enzyme

Lyophilization of enzyme in the presence of a suitable ionic liquid is a simple method to modify the enzyme and can improve enzyme performance in non-aqueous media [32,33,34,35,36,37]. The ionic liquid co-lyophilized enzyme, prepared by different hydrophilic ionic liquids (15%, wt %, IL/enzyme), was used to catalyze the enantioselective esterification of α-lipoic acid. As shown in Table 3, CLL showed poor enzyme performance when CLL was co-lyophilized with [Bmim] Cl or [Bmim] Ac. This may be due to the high hydrogen-bond basicity of these two ionic liquids destroying the enzyme conformation and decreasing the activity and the enantioselectivity. Among the different ionic liquids, [Bmim] BF_4_ was selected as the most suitable ionic liquid for further study since the best catalytic performance was observed when CLL was co-lyophilized with [Bmim] BF_4_. The activation effect of co-lyophilization might be due to the active-site preservation or molecular flexibility increase in organic environments or a combination of both [36,38].

**Table 3:** Effect of ionic liquids on the enantioselective esterification of α-lipoic acid.

Ionic Liquid	Reaction Time (h)	Conversion (%)	*ee*_s_ (%)	Enzyme Activity (μmol/h/mg)	Enantioselectivity (*E* value)
[Bmim] Cl	48	16.8	7.4	0.7	2.3
[Bmim] Ac	30	21.0	11.7	1.4	2.9
[Bmim] BF_4_	5	26.5	27.0	10.6	9.1
None	12	21.6	17.5	3.6	5.3

Reaction conditions: Reaction conditions: (*R*,*S*)-α-lipoic acid (2 mmol), CLL (10 mg) co-lyophilized with different ionic liquid (15%, wt %, IL/enzyme), *n*-octanol (5 mmol) and cyclohexane (10 mL) were performed at 30 °C and 150 rpm.

**Figure 1:**
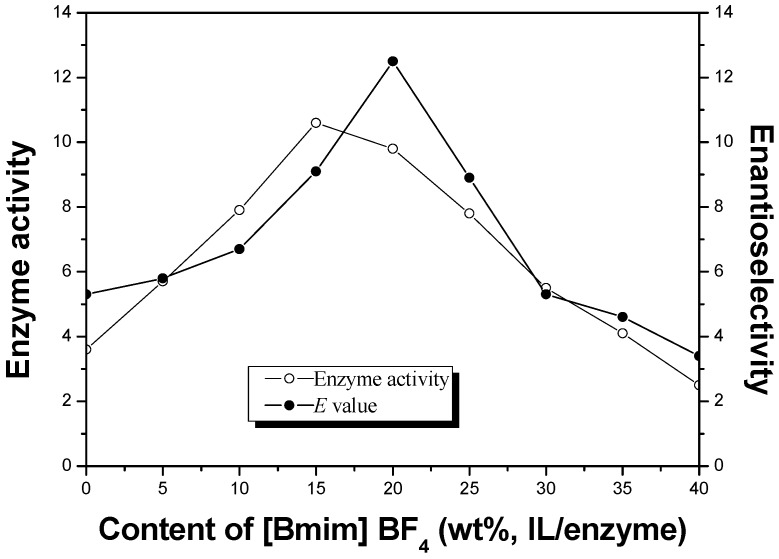
Effects of [Bmim] BF_4_ content during lyophilized preparation on the enzyme activity (μmol/h/mg, ○) and the enantioselectivity (*E* value, ●) in enantioselective esterification of α-lipoic acid. Reaction conditions: (*R*,*S*)-α-lipoic acid (2 mmol), CLL (10 mg) co-lyophilized with different contents of [Bmim] BF_4_ (wt %, IL/enzyme), *n*-octanol (5 mmol) and cyclohexane (10 mL) were performed at 30 °C and 150 rpm.

When the ionic liquid co-lyophilized enzyme catalyzed the enantioselective esterification, the ionic liquid content may be an important influencing factor. In this study, the correlation between the content of [Bmim] BF_4_ (0%–40%, wt %, IL/enzyme) and enzyme performance was investigated. Figure 1 showed that both the enzyme activity and the enantioselectivity exhibited bell-shaped curves when changing the content of [Bmim] BF_4_, which meant a suitable content of [Bmim] BF_4_ was necessary for enzyme lyophilized preparation. The best enzyme performance was obtained at a content of [Bmim] BF_4_ (20%, wt %, IL/enzyme) with an enzyme activity of 9.8 μmol/h/mg and an enantioselectivity (*E* value) of 12.5.

### 2.4. Microwave Irradiation vs. Conventional Heating

Compared with the results obtained from conventional heating, microwave irradiation can enhance both the enzyme activity and enantioselectivity of the free CLL or the ionic liquid co-lyophilized CLL (Table 4). Moreover, the improvement of enzyme performance for the ionic liquid co-lyophilized CLL under MW was much greater than that of the free enzyme. The dramatic enhancement of enzyme performance might be that microwaves can directly act on the ionic liquid co-lyophilized enzyme because of the excellent microwave-absorbing ability of IL [42] and change the protein conformation of CLL.

**Table 4:** Effect of microwaves on the enantioselective esterification of α-lipoic acid.

	Conventional Heating		Microwave	
	Enzyme Activity (μmol/h/mg)	Enantioselectivity (*E* value)	Enzyme Activity (μmol/h/mg)	Enantioselectivity (*E* value)
Free CLL	3.6	5.3	5.1	6.7
Ionic liquid co-lyophilized CLL	9.8	12.5	170.4	38.6

Reaction conditions: (*R*,*S*)-α-lipoic acid (2 mmol), free CLL (10 mg) or CLL (10 mg) co-lyophilized with 20% of [Bmim] BF_4_ (wt %, IL/enzyme), *n*-octanol (5 mmol) and cyclohexane (10 mL) were performed at 30 °C. The microwave was carried out in a microwave bath (480 W). Conventional shaking was performed at 150 rpm.

### 2.5. Effect of Microwave Power

**Figure 2:**
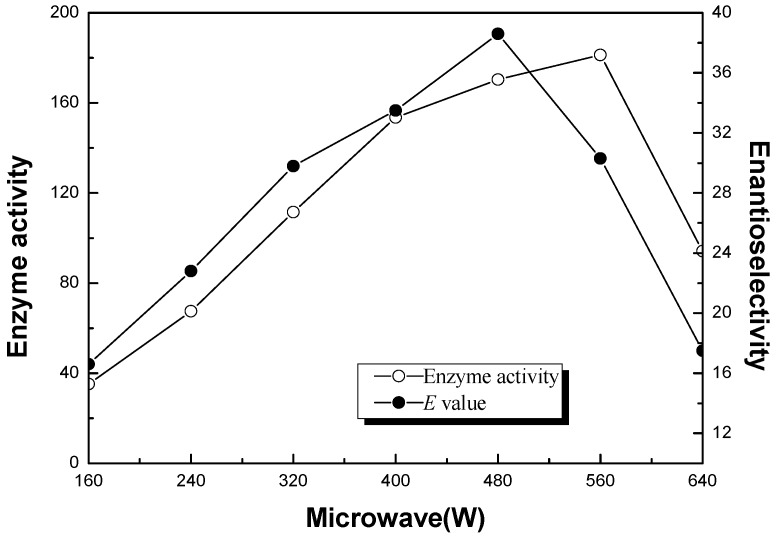
Effect of microwave power on the enzyme activity (μmol/mg/h, ○) and enantioselectivity (*E* value, ●) of ionic liquid co-lyophilized CLL in enantioselective esterification of α-lipoic acid. Reaction conditions: (*R*,*S*)-α-lipoic acid (2 mmol), CLL (10 mg) co-lyophilized with 20% of [Bmim] BF_4_ (wt %, IL/enzyme), *n*-octanol (5 mmol) and cyclohexane (10 mL) were performed at 30 °C under microwave with various power (W).

The effect of microwave power on the enzyme performance of the ionic liquid co-lyophilized CLL was examined in the range of 0–640 W. As shown in Figure 2, ionic liquid co-lyophilized CLL exhibited the highest enantioselectivity (*E* value, 38.6) and a higher enzyme activity (170.4 μmol/h/mg) when the microwave power was 480 W. Generally, the higher the microwave power was set, the faster the dipole reorientated under the MW, which might make the functional groups obtain a higher reactivity [28,43]. Since all of the ionic liquid co-lyophilized enzyme and substrate used in this study had significant dipole moments, microwave could significantly impact theses molecules and activate enzyme and substrate. At high microwave power, the enzyme activity and the enantioselectivity decreased, possibly due to the denaturation of enzymes, caused by the quick change of temperature at the very beginning of the reaction. Based on the experimental results, 480 W was chosen as the optimal microwave power.

### 2.6. Effect of Microwave Temperature

**Figure 3:**
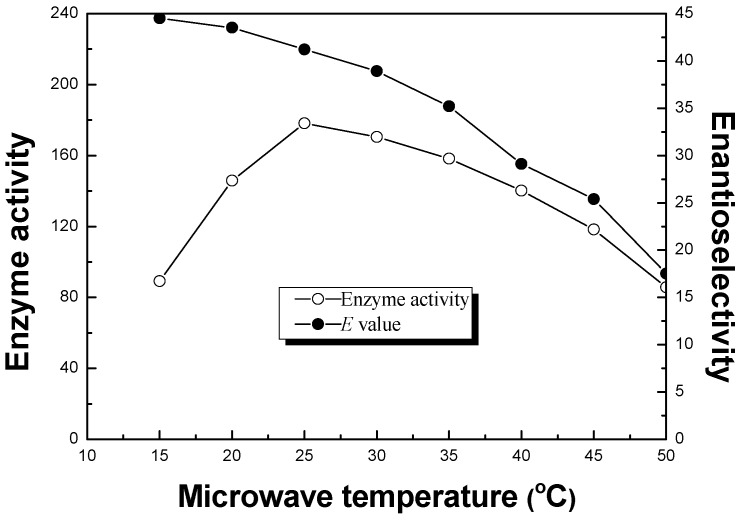
Effect of microwave temperature on the enzyme activity (μmol/mg/h, ○) and enantioselectivity (*E* value, ●) of ionic liquid co-lyophilized CLL in enantioselective esterification of α-lipoic acid. Reaction conditions: (*R*,*S*)-α-lipoic acid (2 mmol), CLL (10 mg) co-lyophilized with 20% of [Bmim] BF_4_ (wt %, IL/enzyme), *n*-octanol (5 mmol) and cyclohexane (10 mL) were performed at different temperature under microwaves (480 W).

The effect of temperature was examined in the range of 15–50 °C under microwave irradiation. The results in Figure 3 demonstrated that the highest enzyme activity (178.1 μmol/h/mg) was obtained, with a high enantioselectivity (*E* = 41.2), at 25 °C for the ionic liquid co-lyophilized lipase. Therefore, 25 °C was selected as the optimal microwave temperature. Under optimal conditions, a preparative reaction on a twenty-fold-larger scale gave (*R*)-α-lipoic acid with about 41% yield and 99% *ee*.

### 2.7. Reusability of the Ionic Liquid Co-Lyophilized Enzyme under Microwave Irradiation

The ionic liquid co-lyophilized lipase exhibited excellent reusability under low power microwaves. As shown in Table 5, the ionic liquid co-lyophilized CLL could still keep 95.2% of its original activity and 94.4% of its original enantioselectivity, even after six reaction cycles.

**Table 5:** Reusability of ionic liquid co-lyophilized CLL under microwaves.

Reaction Cycle	Relative Activity (%)	*E* value
1	100	41.2
2	99.2	40.9
3	98.5	40.6
4	97.7	40.3
5	96.4	39.5
6	95.2	38.9

Reaction conditions: (*R*,*S*)-α-lipoic acid (2 mmol), CLL (10 mg) co-lyophilized with 20% of [Bmim] BF_4_ (wt %, IL/enzyme), *n*-octanol (5 mmol) and cyclohexane (10 mL) were performed at 25 °C under microwaves (480 W).

## 3. Experimental Section

### 3.1. Materials

D-phenylalanine, α-lipoic acid and *o*-phthalaldehyde (OPA) were purchased from Sigma–Aldrich (St. Louis, MO, USA). 11-Mercaptoundecanoic acid was also purchased from Sigma–Aldrich and used as the internal standard. All the ionic liquids (1-butyl-3-methylimidazolium chloride, [Bmim] Cl; 1-butyl-3-methylimidazolium acetate [Bmim] Ac; 1-butyl-3-methylimidazolium tetrafluoroborate, [Bmim] BF_4_) were purchased from Shanghai Chengjie Chemical Co., Ltd. (Shanghai, China). *Candida cylindracea* A.Y. lipase (AYL) was purchased from Amano Pharmaceutical Co., Ltd. (Nagoya, Japan). *Mucor miehei* lipase (MML) was purchased from Novo (Bagsvaerd, Denmark). *Porcine pancreatic* lipase (PPL) was purchased from Shanghai Dongfeng Biochemical Reagent Co., Ltd. (Shanghai, China). *Candida lipolytic* lipase (CLL) was provided by Wuxi enzyme preparation plant (Wuxi, China). All the other chemicals were obtained from commercial sources and were of analytical reagent grade.

The OPA-solution (25 mM) was prepared by dissolving OPA (33.5 mg) in 0.5 mL of methanol and diluted by 10 mL of a potassium borate buffer (0.4 M, pH 9.9). The internal standard solution was prepared by dissolving 10 mg 11-mercaptoundecanoic acid in 10 mL methanol.

### 3.2. Microwave Equipment

Reactions were carried out in a commercial multimode microwave reactor (MCR-3, Shanghai JieSi Microwave Chemistry Corporation). The machine consisted of a continuous focused microwave power delivery system and an operator selectable power output from 0 to 800 W. The temperature of the reaction mixture was monitored and kept constant (±1 °C) by using a contact Teflon platinum resistance temperature transducer inserted directly into the reaction mixture. The content of the vessel was stirred by a rotating magnetic plate located below the base of the microwave cavity and a Teflon-coated magnetic stir bar in the vessel.

### 3.3. Preparation of the Co-Lyophilized Lipase

Lipase powder (1 g) was dispersed in phosphate buffer (50 mL, pH 8.0, 0.1 M) at 4 °C for 2 h under stirring, and the insoluble impurity was removed by centrifugation (8000 rpm, 5 min). The supernatant was lyophilized for 48 h. The lyophilized enzyme powder (200 mg) was dissolved again in the above phosphate buffer (10 mL). To prepare the co-lyophilized lipase, [Bmim] BF_4_ (40 mg) was added to the enzyme solution and the solution was lyophilized for 12 h.

### 3.4. Resolution of α-Lipoic Acid

The reaction mixture containing cyclohexane (10 mL), (*R*,*S*)-α-lipoic acid (2 mmol) and *n*-octanol (5 mmol) was incubated in a microwave oven (480 W, 25 °C, 150 rpm). The reaction was initiated by addition of CLL (10 mg) co-lyophilized with [Bmim] BF_4_ (20%, wt %, IL/enzyme). The reaction mixture (100 μL) was withdrawn periodically and extracted by the solution of sodium bicarbonate (25 mM, 200 μL) to obtain the un-reacted α-lipoic acid aqueous solution for High Performance Liquid Chromatography (HPLC) detection.

### 3.5. Reusability of the Co-Lyophilized Lipase

After each round of reaction, the co-lyophilized lipase was recycled by centrifugation (10,000 rpm, 5 min, 4 °C) and washed with cyclohexane for three times. Then, the enzyme was repeatedly used in the next reaction. The residue activity of the recycled enzyme was compared with the enzyme activity of the first cycle (100%).

### 3.6. High Performance Liquid Chromatography (HPLC) Analysis

α-Lipoic acid was determined using OPA derivatization and fluorescence detection, based on the previous report [44], with a minor modification.

The extracted un-reacted α-lipoic acid solution (50 μL) was mixed with 50 μL of the internal standard solution and 25 μL of a 0.05 M aqueous solution of NaOH containing 0.5 mg/mL of NaBH_4_ and incubated at 60 °C for 10 min to obtain the reduced α-lipoic acid before OPA derivatization. The OPA derivatizations were carried out by mixing the reduced sample solution (25 μL), the d-phenylalanine solution (25 μL, 25 mM) with the OPA-solution (50 μL, 25 mM) for 60 min, standing at room temperature. The reaction was terminated by acidification with 100 µL of phosphate buffer (0.02 M, pH 5.8) before injection.

The sample analysis was performed on an Agilent 1200 HPLC equipped with a 250 × 4 mm LiChrospher 100 RP-18 (5 μm) reversed-phase column (Merck, Darmstadt, Germany). The mobile phase was composed of 55% (*v*/*v*) phosphate buffer (0.02 M, pH 5.8) and 45% (*v*/*v*) acetonitrile/methanol (*v*/*v*, 1:1). A flow rate of 1.8 mL/min was used. Detection was carried out by excitation at 230 nm and emission over 418 nm. The injection of samples (20 μL) was performed using an auto-sampler (Jasco 851-AS).

The enantiomeric excess of the un-reacted α-lipoic acid (*ee*) was determined by calculating the peak areas of the two enantiomers, and the conversion (C) was determined based on the decrease of the substrate. The enzyme activity (μmol/h/mg) was defined as the amount (in micromoles) of the decreased substrate per hour per milligram of enzyme and was detected while the conversion was controlled in the range of the 15%~30%. The enantiomeric ratio (*E* value) was calculated according to Chen *et al*. [45]. All experiments were carried out in triplicate and all data were obtained based on the average values.

(1) $$E=\;\frac{\text{ln}\;\left[\left(1-C\right)\left(1-ee\right)\;\right]}{\text{ln}\;\left[\left(1-C\right)\left(1+ee\right)\;\right]}$$

## 4. Conclusions

In this study, we take the advantage of the synergistic effect between ionic liquid co-lyophilized lipase and microwave irradiation to enhance the enzyme performance in enantioselective esterification of α-lipoic acid. Under the optimal reaction conditions, the ionic liquid co-lyophilized lipase exhibited a satisfied enzyme performance (*E* value, 41.2; enzyme activity, 178.1 μmol/h/mg). Furthermore, high reusability of the ionic liquid co-lyophilized lipase under low power MW conditions make it more attractive for resolution of (*R*,*S*)-α-lipoic acid in industry.
